# Novel targets identified by integrated proteomic and phosphoproteomic analysis in spermatogenesis of swamp buffalo (*Bubalus bubalis*)

**DOI:** 10.1038/s41598-020-72353-4

**Published:** 2020-09-24

**Authors:** Yu-lin Huang, Peng-fei Zhang, Qiang Fu, Weng-tan He, Kai Xiao, Ming Zhang

**Affiliations:** 1 Department of Cell and Genetics, College of Basic Medicine, Guangxi University of Chinese Medicine, Nanning, Guangxi China; 2 State Key Laboratory for Conservation and Utilization of Subtropical Agro-Bioresources, Animal Reproduction Institute, Guangxi University, Nanning, Guangxi China

**Keywords:** Proteomics, Biological techniques, Developmental biology

## Abstract

To understand mechanisms of spermatogenesis, the proteome and the phosphoproteome in prepubertal and pubertal swamp buffalo (*Bubalus bubalis*) testes were analyzed using tandem mass tag (TMT) coupled with liquid chromatography-tandem mass spectrometry (LC–MS/MS). In prepubertal testes, 80 proteins were overexpressed, 148 proteins were underexpressed, and 139 and 142 protein sites had higher and lower phosphorylation, respectively, compared to the levels in pubertal testes. Several of these proteins were associated with reproductive processes such as sexual reproduction, spermatogenesis, fertilization, and spermatid development. In particular, outer dense fiber protein 1 (ODF1), protein maelstrom homolog (MAEL), actin-like protein 7B (ACTL7B), tyrosine-(Y)-phosphorylation regulated (CABYR), and tripartite motif containing 36 (TRIM36) were upregulated with age at both the proteome and phosphoproteome levels. Combining proteome and phosphoproteome analysis can be effectively applied to study the protein/phosphorylation patterns of buffalo testes. These data provide new regulatory candidates and evidence for a complex network in spermatogenesis in buffalo testes, and serve as an important resource for exploring the physiological mechanism of spermatogenesis in mammals.

## Introduction

Spermatogenesis is a complex and dynamic process involving the mitosis of spermatogonia, meiosis of spermatocytes, and spermiogenesis of spermatids eventually^1^. During the process of spermiogenesis, the haploid round spermatids produced by meiosis undergo drastic morphological changes, such as the nuclear formation of the haploid germ cell, the mitochondria are rearranged in a specific manner, the flagellum develops, and the acrosome forms and they become sperm. In mammals, the first wave of spermatogenesis begins after birth and spermatogenesis continues in the seminiferous tubules throughout adulthood, producing huge amounts of sperm after puberty^2^. Spermatogenesis is complex process and is supported by the precise regulation of gene and protein expression. Over the past two decades, advances in molecular biology and genomics have improved our knowledge of spermatogenesis by identifying the genes essential for the development of functional male gametes^3,4^. In addition, the protein expression levels during spermatogenesis have been well studied, and over 7,000 proteins have been identified by high-throughput proteomic studies in mammalian testes^5–7^. However, the dynamic regulatory events that orchestrate this complex process have yet to be elucidated.

Phosphorylation, a ubiquitous and important post-translational modification (PTM), is one of the most critical regulatory mechanisms of the cell cycle^8^, which is particularly active during spermatogenesis. A number of significant studies have contributed to our understanding of the regulation of phosphorylation in spermatogenesis. For instance, TH2B, an important testicular-specific histone, acts as a ‘phosphorylation switch’ and plays a vital role in chromatin structural remodeling of spermatogenesis^9^. Testis specific serine kinase 4 (TSSK4) belongs to the testis specific serine/threonine protein kinase (TSSK) family and is required for maintaining the structural integrity of sperm flagellum and male fertility^10^. Mitogen-activated protein kinase (ERK1/2) plays an important role in ectoplasmic specialization dynamics during spermatogenesis^11^. Hence, systematic analysis of testicular phosphorylation is also important for understanding the molecular mechanism of spermatogenesis. In recent years, proteomics techniques based on mass spectrometry have evolved rapidly to identify thousands of PTM sites in a single run^12^. However, only a few studies have contributed to the identification of the phosphoproteome in testes^13–16^. Huttlin et al. identified about 36,000 phosphorylation sites in 6,296 proteins from nine tissues (including testicles) of 3-week-old immature mice^13^. Shi et al. established the testicular phosphoproteome in perfluorododecanoic acid (PFDoA)-exposed rats, and discovered 937 unique phosphorylation sites^14^. Qi et al*.* performed a systematic analysis of the phosphoproteome in mouse testes and identified a total of 17,829 phosphorylation sites^15^. A recent study identified a total of 8,187 phosphopeptides derived from 2,661 proteins in human testes^16^. Nevertheless, there have been no reports on the buffalo testicular phosphoproteome.

The swamp buffalo (*Bubalus bubalis*) is of considerable economic and biological interest among large farm animals, especially for tropical and subtropical regions, but its reproductive efficiency is low compared to other domesticated ruminants^17^. Thus, a better understanding of the molecular mechanisms of spermatogenesis in this ruminant species will be valuable. Differential proteomic analysis is a reliable and reproducible high-throughput approach to study the molecular mechanisms of spermatogenesis^18^. Protein phosphorylation plays an important role in cell division, proliferation, differentiation, metabolism, and is closely related to spermatogenesis^15^. There has been no study on the quantitative changes in proteomics and protein phosphorylation in prepubertal and pubertal buffalo (*Bubalus bubalis*) testes to date, and a comparative proteomic and protein phosphorylation analysis was therefore performed. The results elucidate the mechanisms of spermatogenesis and offer a new perspective for future research into male mammalian reproduction.

## Results

### Differential proteome analysis

**Figure 1:**
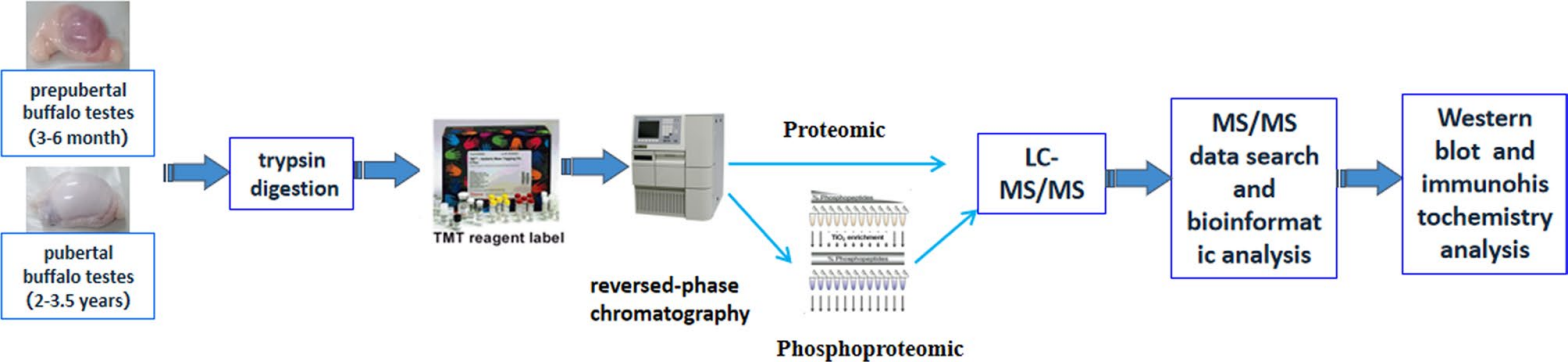
Overview of experimental procedures used in this study.

**Figure 2:**
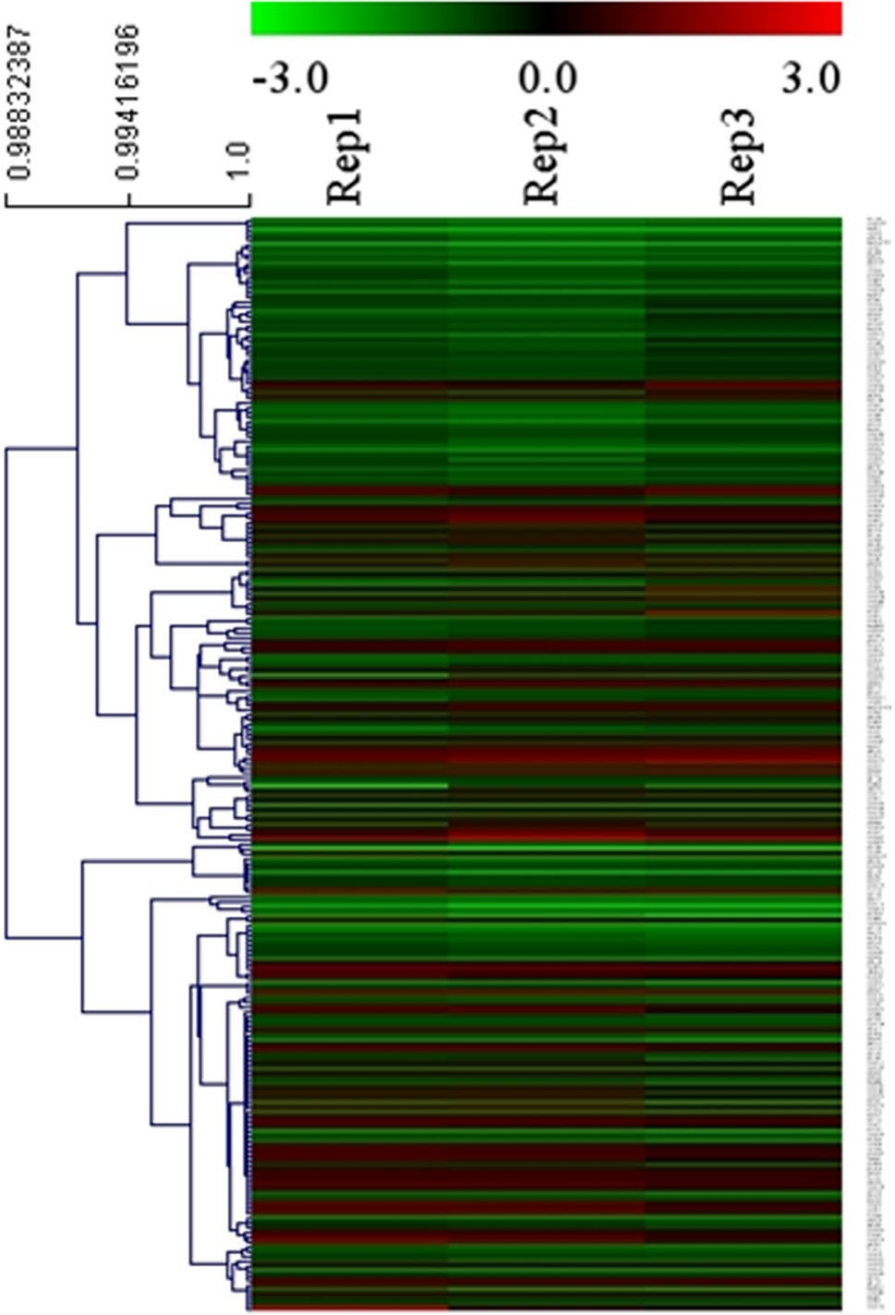
Hierarchical clustering analysis of the differentially expressed proteins. The columns and rows represent experiment repetition and differential proteins, respectively. The color represents the expression level of differential proteins (log2), red represents the up-regulated proteins, and green represents the down-regulated proteins.

An overview of the experimental procedures used in this study is shown in Fig. 1. Here, 3,966 proteins were identified, and 3,944 were quantified. With a fold-change threshold of > 1.5 and a P value < 0.05, 80 and 148 proteins were up- and down-regulated in prepuberty/puberty, respectively. Distinct and reproducible patterns of statistically significant changes in protein expression were detected among three independent experiments (Rep1, Rep2, and Rep3) (Fig. 2; Supplementary Table S1).

**Figure 3:**
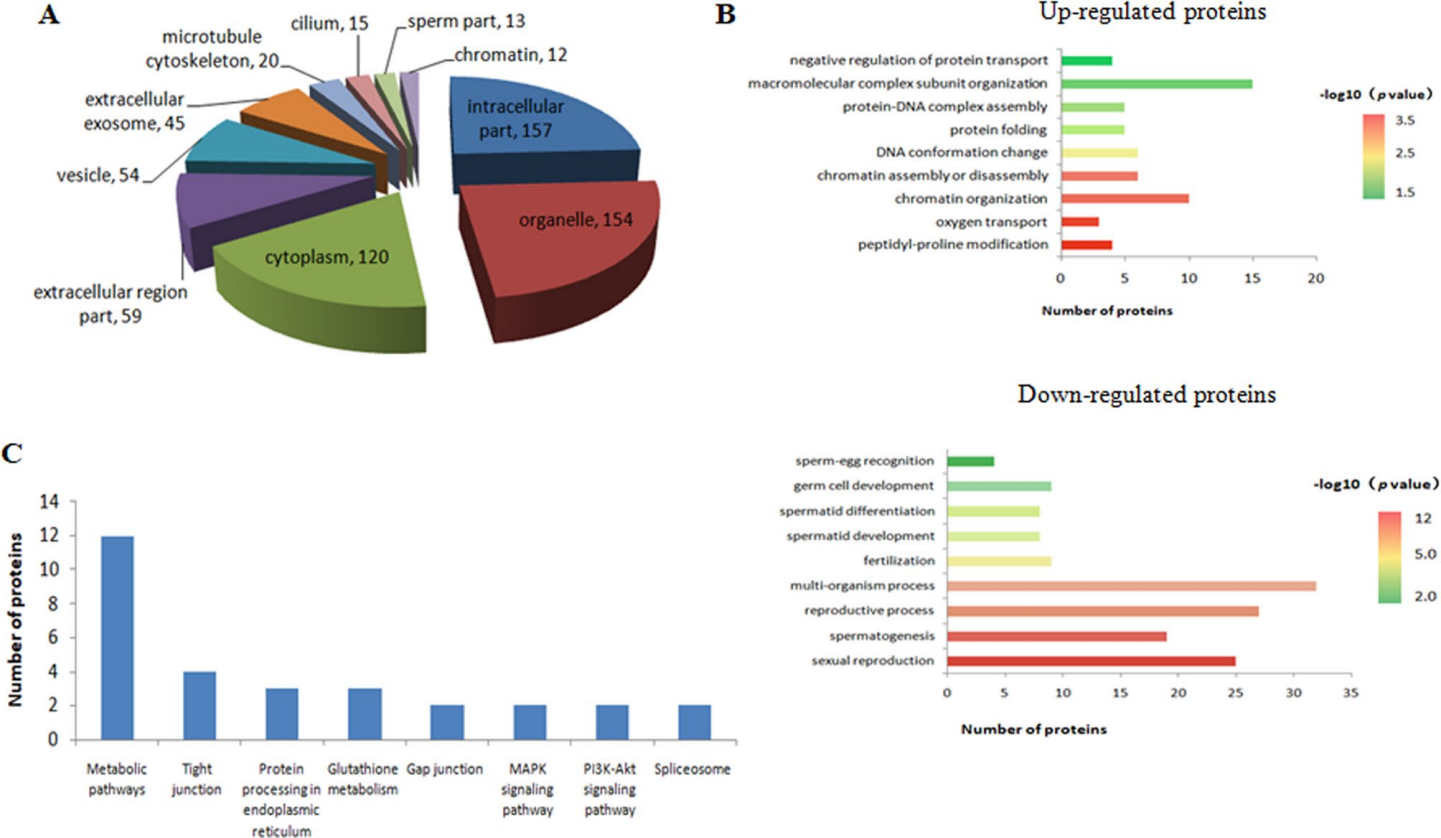
Functional annotation of the identified differential expression proteins. (**A**) Subcellular classification. (**B**) Biological process category. (**C**) KEGG pathway analysisfor all differentially expressed proteins.

All differentially expressed proteins (DEPs) were located in the intracellular parts, organelles, cytoplasm, extracellular regions, vesicles, extracellular exosomes, microtubule cytoskeleton, cilia, sperm, and chromatin, according to the subcellular classification (Fig. 3A). Up-regulated proteins in the biological process category were macromolecular complex subunit organization, chromatin organization, chromatin assembly or disassembly, DNA conformation change, and protein folding. Down-expressed proteins participated in multi-organism processes, reproductive processes, sexual reproduction, spermatogenesis, fertilization, and spermatid development (Fig. 3B). In pubertal buffalo (*Bubalus bubalis*) testes, a large number of DEPs may be involved in spermatogenesis.

The cellular pathways of the DEPs were profiled using the Kyoto Encyclopedia of Genes and Genomes (KEGG) database. Enrichment of several pathways involved in metabolic pathways, tight junction, protein processing in endoplasmic reticulum, glutathione metabolism, gap junction, MAPK signaling pathway, and spliceosome were observed (Fig. 3C). It has been reported that the tight junction between Sertoli cells and germ cells plays an important regulatory role in spermatogenesis^19,20^. In this study, four DEPs, GATA binding protein 4 (GATA4), Na^+^/H^+^ exchange regulatory cofactor NHE-RF1 (SLC9A3R1), tubulin alpha-8 chain (TUBA8), and CSDA, were involved in the tight junction pathway (Supplementary Figure S1).

### Differential phosphoproteome analysis

**Figure 4:**
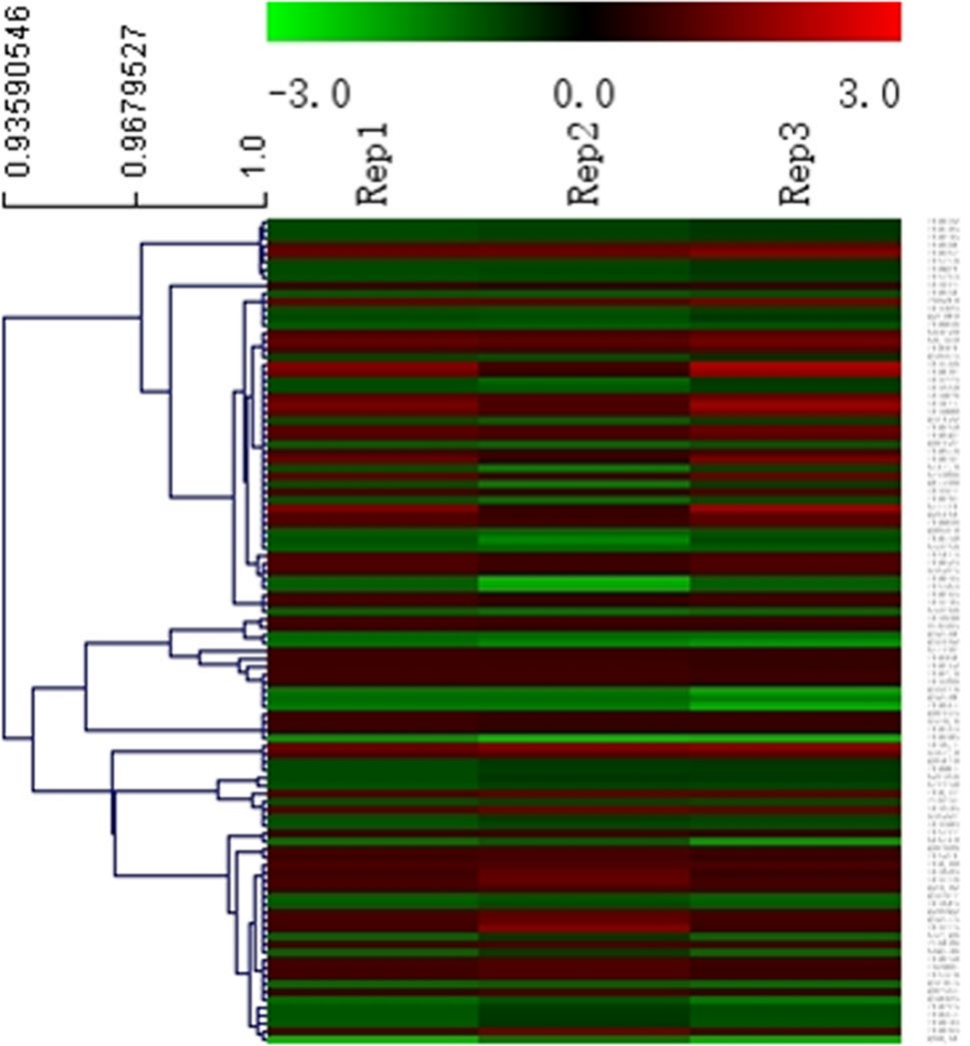
Hierarchical clustering analysis of the differentially expressed phosphoproteins. The columns and rows represent experiment repetition and differential phosphoproteins, respectively. The color represents the expression level of differential phosphoproteins (log2), red represents the up-regulated phosphoproteins, and green represents the down-regulated phosphoproteins.

A total of 3,049 phosphosites in 689 proteins were identified, and 3,032 phosphosites in 686 proteins were quantified according to the LC–MS/MS analysis. According to a fold-change threshold of > 1.5 and a P value < 0.05, 139 phosphosites in 56 proteins and 142 phosphosites in 48 proteins were up- and down-regulated in prepuberty/puberty, respectively. Distinct and reproducible patterns of statistically significant changes in protein phosphorylation were detected in three independent experiments (Rep1, Rep2, and Rep3) (Fig. 4; Supplementary Table S2).

**Figure 5:**
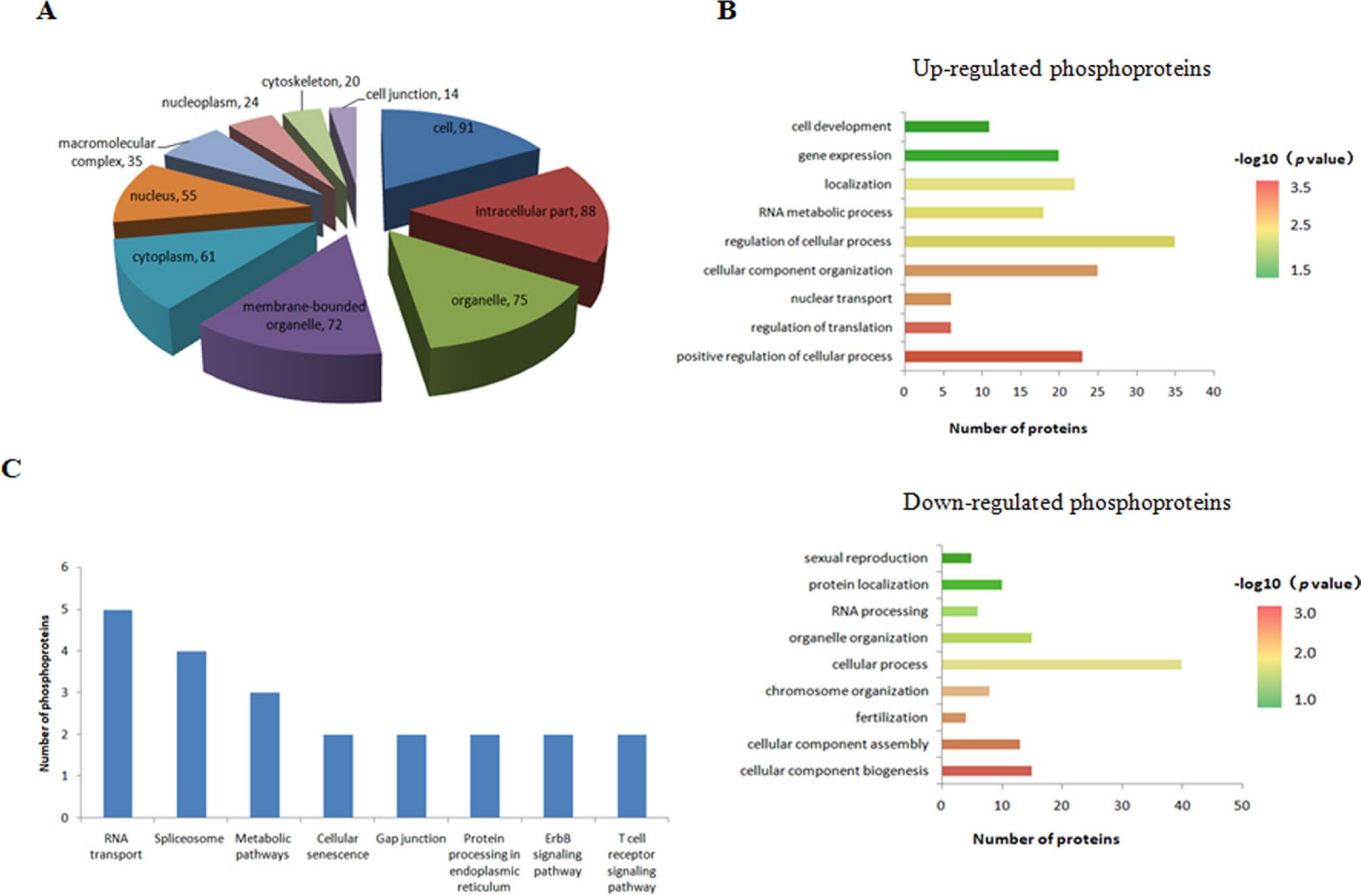
Functional annotation of the identified differential expression phosphoproteins. (**A**) Subcellular classification. (**B**) Biological process category. (**C**) KEGG pathway analysisfor all differentially expressed phosphoproteins.

The differential expression phosphoproteins (DEPPs) were mainly located in the cell, intracellular parts, organelles, membrane-bounded organelles, cytoplasm, macromolecular complex, nucleoplasm, cytoskeleton, and cell junction according to the subcellular classification (Fig. 5A). In the biological process category, regulation of cellular processes, cellular component organization, positive regulation of cellular processes, localization, gene expression, RNA metabolic processes, and cell development were highly enriched among the up-regulated phosphoproteins. Down regulated phosphoproteins were mainly involved in cellular processes, cellular component biogenesis, organelle organization, cellular component assembly, chromosome organization, and sexual reproduction (Fig. 5B).

Enrichment of several pathways involved in RNA transport, spliceosome, metabolism, cellular senescence, gap junctions, protein processing in the endoplasmic reticulum, ErbB signaling, and T cell receptor signaling were identified using the KEGG database (Fig. 5C). Spliceosomal components are involved in the formation of male gametes, and major spliceosome defects cause male infertility^21,22^. In this study, four DEPPs, including nuclear cap binding protein subunit 1 (NCBP1), splicing factor 3b subunit 1(SF3B1), splicing factor 3A subunit 1 (SF3A1), and serine/arginine-rich splicing factor 2 (SRSF2), were involved in the spliceosome pathway (Supplementary Figure S2).

### Integrated analysis and validation of proteome and phosphoproteome data

**Figure 6:**
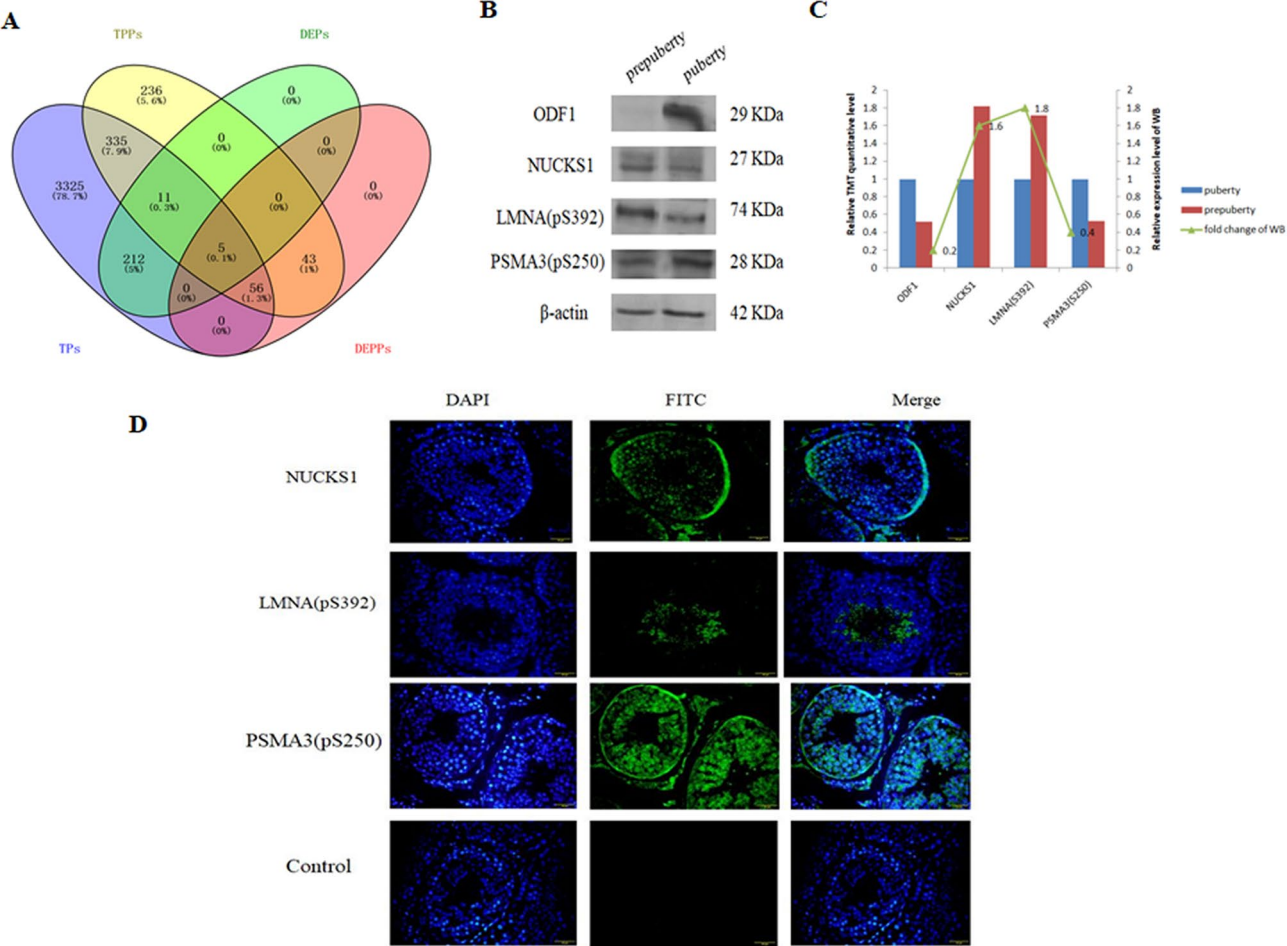
Integratedanalysis and validation of proteome and phosphoproteome data. (**A**)The Venn diagram for identified total proteins (TPs), phosphoproteins (TPPs), differential expression proteins(DEPs) and differential expression phosphoproteins (DEPPs) of prepubertal and pubertal buffalo(*Bubalus bubalis*) testes. (**B**) Validation of proteome and phosphoproteome results by Western blot analysis. ODF1 and PSMA3 (pS250) were up-regulated, NUCKS1 and LMNA (pS392) were down-regulated in puberty in the cropped gels. β-actin was used as loading control. Full-length gels are presented in Supplementary Figure S3. (**C**) Combination figure of TMT quantitative data and Western blot analysis results. (**D**) Immunohistochemical analyses of NUCKS1, LMNA (pS392), and PSMA3 (pS250) in pubertal buffalo testis. NUCKS1 was mainly expressed in the nucleus of spermatogonium and primary spermatocyte, LMNA (pS392)only located in the lumen of seminiferous tubule, whereas PSMA3 (pS250)was highly expressed in the cytoplasm and nucleus of spermatogenic cells, Sertoli cells, and leydig cells of buffalo testis. Scale bars = 50 μm.

The analysis of proteome and phosphoproteome data was integrated and 407 proteins were found in total quantitative proteins (TPs, 3,944) and total quantitative phosphoproteins (TPPs, 686). There were 16 DEPs with phosphosites, five of which (ODF1, MAEL, ACTL7B, CABYR, and TRIM36) were also DEPPs. Interestingly, the five proteins were upregulated at both the proteome and phosphoproteome levels in pubertal buffalo (*Bubalus bubalis*) testes (Fig. 6A; Table 1).

**Table 1:** List of 5 up-regulated proteins with age at both the proteome and phosphoproteome levels.

Accession	Description	Gene	Prepuperty/puperty (proteome)	Modifications	Prepuperty/puperty (phosphoproteome)
E1BAW5	Tripartite motif containing 36	TRIM36	0.65	Y613; S617; S621; S623	0.5
Q32L61	Calcium binding tyrosine-(Y)-phosphorylation regulated	CABYR	0.63	S331; S335	0.43
Q32L91	Actin-like protein 7B	ACTL7B	0.61	S6; S7; S8	0.40
F1MMI4	Protein maelstrom homolog	MAEL	0.56	S95	0.59
F1MZM5	Outer dense fiber protein 1	ODF1	0.52	S32; S64; S167	0.26

Outer dense fiber protein 1 (ODF1) and nuclear ubiquitous casein and cyclin-dependent kinase substrate 1 (NUCKS1) from proteome data and phosphosite (Ser392) of lamin A/C (LMNA, pS392) and phospho-site (Ser250) of proteasome subunit alpha type-3 (PSMA3, pS250) from phosphoproteome data were confirmed by western blot (WB) analysis. The results of WB analysis of four proteins (ODF1, NUCKS1, LMNA (pS392), and PSMA3 (pS250)) were consistent with the LC–MS/MS data (Fig. 6B,C). Three candidate proteins (NUCKS1; LMNA, pS392; and PSMA3, pS250) were further investigated by immunohistochemistry analysis (Fig. 6D). NUCKS1 was expressed in the nucleus of spermatogonium and primary spermatocytes, LMNA (pS392) was only located in the lumen of seminiferous tubules, whereas PSMA3 (pS250) was highly expressed in the cytoplasm and nucleus of spermatogenic cells, Sertoli cells, and Leydig cells of buffalo (*Bubalus bubalis*) testes.

## Discussion

In this study, 228 DEPs (80 and 148 proteins were up- and down-regulated in prepuberty, respectively) were successfully identified by TMT labeling combined with LC–MS /MS technology, and were then annotated to specific cell processes based on the results of a bioinformatics analysis. In previous studies, the focus has been on the seminiferous tubules of buffalo testes, and not the whole testicle^23,24^. In addition, traditional two-dimensional electrophoresis coupled to tandem mass spectrometry was used to explore the different proteins in developmental stages of swamp buffalo testicular seminiferous tubules, and only 13 differentially expressed proteins were identified^24^. There are few proteomic analyses of testes in farm animals. Tripathi et al*.* established the differential proteomic profile of spermatogenic and Sertoli cells in crossbred and purebred bulls by 2-dimensional difference gel electrophoresis (2D-DIGE) coupled with mass spectrometry, and only a dozen different proteins were identified^25^. Therefore, the current study is of significance in the field of livestock proteomics.

In this study, up-regulated proteins were mainly involved in protein processing, modification, and folding, while biological processes such as spermatogenesis and development were highly enriched among the downregulated proteins. The histone family members, including H1F0, HIST2H2AB, HIST2H2AC, HIST1H2BJ, HIST2H3D, and HIST1H4C, were all upregulated in prepubertal buffalo (*Bubalus bubalis*) testes, playing an important role in gene transcriptional regulation, DNA repair, DNA replication, and chromosome formation. In addition, several studies have reported the importance of histone modification in spermatogenesis^26–29^. Hemoglobin HBA and HBB were also upregulated in prepubertal buffalo (*Bubalus bubalis*), mainly for oxygen and carbon dioxide transport. NUCKS1 is usually overexpressed in many cancer tissues^30^. However, NUCKS1 was upregulated in prepubertal buffalo (*Bubalus bubalis*) testes and is mainly expressed in the nucleus of spermatogonia and primary spermatocytes. The other up-regulated proteins in prepubertal buffalo (*Bubalus bubalis*) testes include nucleoside diphosphate kinase B (NME2), peptidyl-prolyl cis–trans isomerase C (PPIC), peptidyl-prolyl cis–trans isomerase FKBP9, protein phosphatase 1 regulatory subunit (PPP1R3D), secernin-1 (SCRN1), steroid 17-alpha-hydroxylase/17,20 lyase (CYP17A1), thioredoxin (TXN), sulfatase-modifying factor 1 (SUMF1), and tubulin beta-2B chain (TUBB2B).

Heat shock-related 70 kDa protein 2 (HSPA2) and ropporin-1 (ROPN1) were downregulated in prepubertal buffalo (*Bubalus bubalis*) testes, which concurred with previous results in this laboratory^18^. Many studies have also shown that HSPA2 plays a key role in spermatogenesis and male reproduction^31,32^. A deficiency of ROPN1 protein in mouse sperm can significantly reduce fertility^33^. Sperm surface protein Sp17 (SPA17) is specifically expressed in the testes and sperm, was and is the zona pellucida binding protein on the sperm surface and contributes to the high affinity binding between sperm and the zona pellucida^34,35^. SPA17 was highly expressed in pubertal buffalo (*Bubalus bubalis*) testes, as was tubulin (including TUBA8, TUBB3, and TUBB4A). Tubulin is the major constituent of microtubules and is involved in the formation of testicular seminiferous tubules, and tubulin is involved in spermatogenesis^36–38^. It has been discovered that the expression of outer dense fiber protein 1 (ODF1) is significantly decreased in the ejaculated spermatozoa of asthenozoospermic men, which might be responsible for low sperm motility^39^. The average downregulated ratio of ODF1 expression was 0.53 in prepubertal buffalo (*Bubalus bubalis*) testes, which was confirmed by westernblotting. Tektin-5 (TEKT5), testis-expressed sequence 30 protein (TEX30), testis expressed 101(TEX101), transthyretin (TTR), transitional endoplasmic reticulum ATPase (VCP), T-complex protein 1 subunit zeta-2 (CCT6B), and profilin-3 (PFN3) were downregulated in prepubertal buffalo (*Bubalus bubalis*) testis.

**Figure 7:**
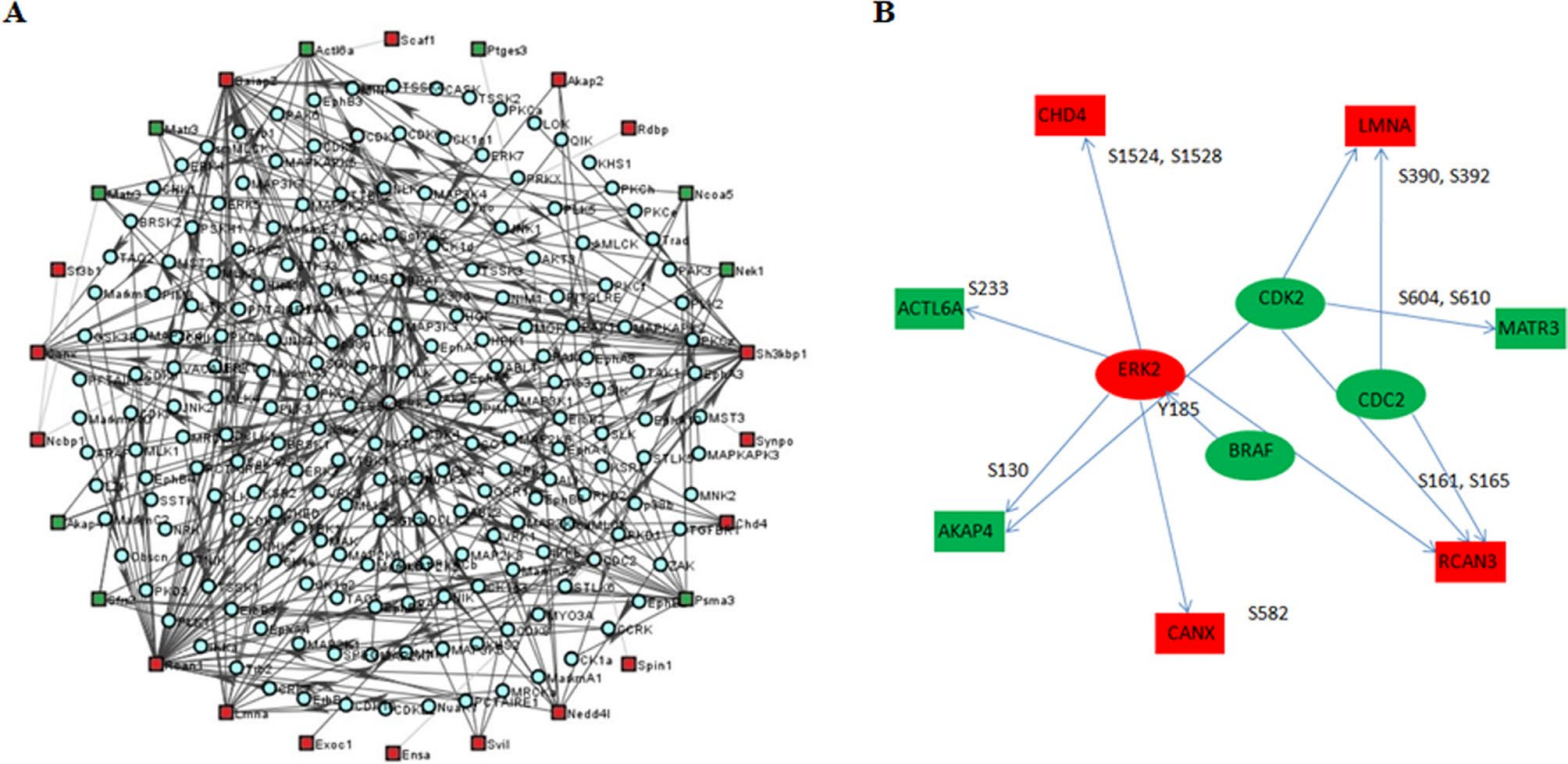
Network analysis of phosphorylation regulation in the buffalo testis phosphoproteome. (**A**) Kinase-substrate network of all differential phosphorylation sites. (**B**) Kinase-substrate networks of four kinases identified in this study. Circle represents kinase, square represents substrate, red represent the up-regulated proteins and green represent the down-regulated proteins in prepubertal buffalo(*Bubalus bubalis*) testis.

Subsequently, 281 phosphosites in 104 DEPPs (139 phosphosites in 56 proteins and 142 phosphosites in 48 proteins were up- and down-regulated in prepubertal buffalo (*Bubalus bubalis*), respectively) using TMT coupled to LC–MS/MS. Through Gene Ontology (GO) analysis, it was found that up-regulated phosphoproteins were mainly involved in the regulation of cellular processes and post-translational modifications, while fertilization, reproduction, and chromosome assembly were highly enriched among the down-regulated phosphoproteins, which is consistent with the biological process of the proteome. In the phosphoproteome, LMNA (pS392) was upregulated in prepubertal buffalo (*Bubalus bubalis*) and was located in the lumen of the seminiferous tubule by immunohistochemistry analysis. It has been shown that Lmna-deficient mice display impaired spermatogenesis^40^. Phospho-sites (Ser392 and Ser390) of LMNA were found to be substrates of CDC2 and CDK2 kinases by kinase-substrate network analysis (Fig. 6).CDC2 and CDK2 are members of cyclin-dependent protein kinase (CDK), and the effect of LMNA phosphorylation on spermatogenesis can be verified using a CDK inhibitor. Through kinase-substrate network analysis, phospho-site (Ser250) of PSMA3 was predicted to be a substrate of POLO-like kinases (PLKs, including PLK1, PLK2, PLK3, PLK4, and PLK5) and casein kinase 1 (CK1, including CK1a, CK1d, CK1e, CK1g1, CK1g2, CK1g3, TTBK1, TTBK2, VRK1, VRK2, and VRK3) (Fig. 7). As important regulators of the cell cycle, PLKs, especially PLK1, were found to be required in multiple stages of spermatogenesis^15,41,42^. Furthermore, it was observed that spermiogenesis initiation in *Caenorhabditis elegans* involves CK1^43^. In this study, PSMA3 (pS250) was highly expressed in the cytoplasm and nucleus of spermatogenic cells, Sertoli cells, and Leydig cells of pubertal buffalo (*Bubalus bubalis*), and the effect of PSMA3 (pS250) on spermatogenesis using PLKs and CK1 inhibitors can be investigated further.

Through integrated analysis of proteome and phosphoproteome data, five proteins (ODF1, MAEL, ACTL7B, CABYR, and TRIM36) were upregulated at both the proteome and phosphoproteome levels in pubertal buffalo (*Bubalus bubalis*) testes. ODF1 protein levels are elevated in puberty, as well as the activating phosphorylation at Ser32, Ser64, and Ser167^23^. However, no commercially available phospho-antibodies are available, and ODF1 phosphorylation sites could not be identified by LC–MS/MS analysis. MAEL is indispensable for spermatogenesis and transposon repression in meiosis^44^. In this study, the phospho-site Ser95 of MAEL changed with the protein level. ACTL7B, a member of the actin family, was highly expressed in pubertal buffalo (*Bubalus bubalis*) at the protein level and phosphorylation at Ser6, Ser7, and Ser8. T-ACTINs play important roles in sperm function and in the specific morphogenesis of spermatozoa during spermiogenesis^45^. TRIM36 plays a crucial role in the arrangement of somites during *Xenopus* embryogenesis^46^, but no studies have reported this in mammals. Phospho-sites (Ser617, Ser621, Ser623, and Tyr613) of TRIM36 and protein level were upregulated in pubertal buffalo (*Bubalus bubalis*) testes. Previous studies have demonstrated that CABYR was involved in capacitation and the phosphorylation of tyrosine and serine/threonine occurs frequently during sperm capacitation in vitro^47,48^. CABYR is expressed in late steps of spermiogenesis and bound to AKAPs and ropporin is expressed in the sperm fibrous sheath^49^. The protein level of CABYR increased with the development of buffalo (*Bubalus bubalis*) testicles and phospho-sites Ser331 and Ser335.were activated. This indicates that the development of buffalo (*Bubalus bubalis*) testicles is regulated by the phosphorylation of CABYR.

In conclusion, the data revealed that reliable TMT-LC–MS/MS-based analyses of both the proteome and the phosphoproteome can be successfully applied to pre- and pubertal buffalo (*Bubalus bubalis*), yielding distinct and reproducible patterns of differentially expressed and phosphorylated genes. In the prepubertal testes, 80 proteins were overexpressed, and 148 proteins were underexpressed; and 139 and 142 protein sites had higher and lower phosphorylation, respectively, as compared to pubertal testes. These findings provide new promising candidate genes and an indication of the complex network of synergistically acting and cooperating mechanisms occurring during the development of buffalo (*Bubalus bubalis*) testes, especially in spermatogenesis. The research provides a resource for further studies on spermatogenesis, while elucidating the mechanisms of regulation of male reproduction buffalo (*Bubalus bubalis*) and by extrapolation, mammals.

## Material and methods

### Sample preparation and protein extraction

Sample preparation and protein extraction were performed using the procedure described previously^18^, with minor modification. Swamp buffalo (*Bubalus bubalis*) testes were collected from a local commercial slaughterhouse and transported to the lab in sterile isotonic saline within 4 h, and the tunica vaginalis and epididymis were removed. Three testes of 2–5 g weight were harvested from prepubertal buffalo (*Bubalus bubalis*) (3–6 months of age), and three testes weighing 32–40 g were harvested from pubertal buffalo (*Bubalus bubalis*) (2–3.5 years of age). A section of one pubertal buffalo (*Bubalus bubalis*) testis was used for immunohistochemistry analysis. The six collected testes were divided into three separate samples to obtain three biological replicates. The weight and length of each testis was recorded (Table S3). All procedures involving animal treatment and collection of testes to be used in the study were based on the Guiding Principles for Animal Use as described by the Council for International Organizations of Medical Sciences (CIOMS) and approved by the Animal Experimentation Ethics Committee of Guangxi University, Nanning, China.

The buffalo (*Bubalus bubalis*) testicular tissue was dissected into small pieces and ground to powder in liquid nitrogen and then transferred to a 5 mL centrifuge tube. Four volumes of lysis buffer (8 M urea, 2 mM EDTA, 1% protease inhibitor cocktail and 1% phosphatase inhibitor cocktail) were added to the cell powder, followed by sonication three times on ice using a high-intensity ultrasonic processor (Scientz, Ningbo, Zhejiang, China). The remaining debris was removed by centrifugation at 12,000×*g* at 4 °C for 10 min. The supernatant was collected and the protein concentration was determined with a BCA kit (Solarbio, Beijing, China), according to the manufacturer’s instructions.

### Trypsin digestion, TMT labeling, and fractionation

The methods described below have been reproduced in part from a previous study^18^. For digestion, 100 μg of protein was reduced with 5 mM dithiothreitol (DTT) for 30 min at 56 °C and alkylated with 11 mM iodoacetamide for 15 min at room temperature in the dark. The protein sample was then diluted by adding 100 mM NH_4_HCO_3_ to a urea concentration of less than 2 M. Trypsin was added to the diluted protein sample at a 1:50 trypsin-to-protein mass ratio for the first digestion overnight and a 1:100 trypsin-to-protein mass ratio for a second digestion for 4 h. The peptides thus obtained were quantified by Pierce Quantitative Fluorometric Peptide Assay (Thermo Scientific, Waltham, MA, USA) and were then labeled with TMT reagents (Thermo Fisher Scientific, Massachusetts, USA) according to the manufacturer’s instructions. In brief, 41 μL of anhydrous acetonitrile (ACN) was added to each tube (0.8 mg), and the sample was dissolved by vortexing for 5 min at room temperature. Each sample was labeled by adding 20 μL of tag and incubating for 1 h at room temperature. Then, 8 μL of 5% hydroxylamine was added to each sample and the sample was incubated for 15 min to quench the reaction. All labeled samples were pooled and evaporated in a vacuum.

The labeled peptide mixtures were suspended in solvent-A (2% ACN, pH 10.0) and separated by reversed-phase chromatography in an HPLC system (Waters, e6295) at a flow rate of 0.5 mL/min. The following gradient was employed: 100% A for 10 min; 0–35% B (98% ACN, pH 10.0) for 45 min; 35–100% B for 10 min, and 0% B for 15 min before the next run. The elution time was 80 min, and the components within 10–65 min were collected every 2 min and combined into 12 fractions, evaporated under vacuum, and then desalted using a ZipTips C18 column (Millipore, Billerica, MA, USA).

### Phosphopeptide enrichment

The phosphopeptide was enriched by TiO_2_, as previously described by Gupta et al.^50^. A total of 4 mg of protein from each sample was subjected to in-solution trypsin digestion, and the peptides that were obtained were quantified by the Pierce quantitative fluorometric peptide assay. A total of 3 mg of peptides from each sample was desalted using Sep-Pak Classic C18 Columns (Waters, Milford, Massachusetts, USA) and phosphopeptide enrichment was done using a High-Select TiO_2_ phosphopeptide enrichment kit (Thermo Scientific, Waltham, MA, USA) following the recommended protocol. Briefly, desalted lyophilized peptides were dissolved in the binding/equilibration buffer provided with the kit and centrifuged to be clarified the dissolved peptides.TiO_2_ spin tips were washed twice with wash buffer and equilibrated once with binding/equilibration buffer before loading of peptides. Phosphopeptides were allowed to bind to the TiO_2_ resin followed by sequential washing with binding buffer and wash buffer. Finally, bound phosphopeptides were eluted using elution buffer and lyophilized quickly to avoid dephosphorylation. The obtained phosphopeptides were labeled with TMT reagents according to the manufacturer’s instructions.

### LC–MS/MS analysis

The LC–MS/MS method has been reproduced in part by Gupta et al.^50^. Lyophilized peptides were dissolved in buffer A (2% ACN and 0.1% formic acid) and separated by reversed-phase chromatography using an Easy-nLC 1000 nano liquid chromatography system (Thermo Fisher Scientific, Odense, Denmark).The sample was continuously separated on an Acclaim PepMap 100 capillary column (75 μm × 15 cm, Nano Viper C18, 3 μm, 100 Å) at a flow rate of 300 nL/min. The LC analytical gradient was run at 5% to 35% buffer B (98% ACN and 0.1% formic acid) over 45 min, then 35% to 100% over 10 min, and finally 100% buffer B for 5 min. Liquid chromatography-tandem mass spectrometry (LC–MS/MS) was coupled with an electrospray ionization source to the quadrupole-based mass spectrometer Q Exactive (Thermo Scientific, Waltham, MA, USA). The electrospray voltage applied was 2.0 kV. The m/z scan range was 300–1,800 for the full scan, and intact peptides were detected in the Orbitrap at a resolution of 70,000. Peptides were then selected for MS/MS using an NCE setting of 27, and the fragments were detected in the Orbitrap at a resolution of 17,500.A data-dependent procedure alternated between one MS scan followed by 20 MS/MS scans with 45 s dynamic exclusion. Automatic gain control (AGC) was set at 5E4.

### MS/MS data search

The method of MS/MS data search has been reproduced in part by Huang et al.^51^. The resulting MS/MS data were processed using the Maxquant search engine (v.1.5.2.8). Tandem mass spectra were searched against the Proteomes *Bos taurus* (24,215 sequences) database concatenated with a reverse decoy database. Trypsin specificity was required, and a maximum of 2 missed cleavages was allowed. The mass tolerance for precursor ions was set as 20 ppm in the first search and 5 ppm in the main search, and the mass tolerance for fragment ions was set as 0.02 Da. Carbamidomethyl on Cys was set as a fixed modification, while TMT-labeled N-term, oxidation on Met and protein N-terminal acetylation were set as variable modifications in case of proteome analysis, and the phosphorylation of Ser, Thr, and Tyr residue (phosphorSTY) was added as a variable modification in case of phosphoproteome analysis. The false discovery rate (FDR) was adjusted to < 1% and phosphopeptides that were reproducibly identified in at least two out of three replicates of at least one sample with score > 40, and where the site localization probability was set as > 0.75, were considered as valid identification and used for further analysis.

### Bioinformatic analysis

Bioinformatics analysis was performed according to our previous study^18^, with minor modifications. The GO annotation proteome was derived from the UniProt-GOA database, and the proteins were classified by GO annotation involving three categories: biological processes, molecular functions, and cellular components using the bioinformatics tool DAVID v6.8. The SpermatogenesisOnline database (https://mcg.ustc.edu.cn/bsc/spermgenes/documentation.php) was used to analyze and verify spermatogenesis-related process terms. The Kyoto Encyclopedia of Genes and Genomes (KEGG) database was used to perform pathway analysis, and the iGPS software package^52^ (https://igps.biocuckoo.org) was used to predict potential site-specific kinase-substrate relations.

### Western blot analysis

Western blot analysis was performed as described previously^18^ with a modification. Equal amounts of proteins were separated by SDS-PAGE and transferred onto a polyvinylidene fluoride (PVDF) membrane using a semidry western blotting system (Bio-Rad).The membrane was blocked with 5% nonfat milk in Tris Buffered Saline Tween (TBST) solution for 2 h at room temperature and then incubated overnight at 4 °C with primary antibody against ODF1 (ab197029, Abcam, Cambridge, MA, USA), NUCKS1 (bs-7917R, Bioss Biotechnology Inc., Beijing, China), LMNA (phospho S392) (ab58528, Abcam), PSMA3 (phospho S250) (bs-9353R, Bioss Biotechnology Inc.), and β-actin (loading control) (bs-0061R, Bioss Biotechnology Inc.) at a dilution of 1:1,000. Membranes were washed with TBST buffer three times and incubated with horseradish peroxidase-conjugated secondary antibodies (CWBIO, Beijing, China) in TBST buffer for 1.5 h at room temperature. Bands were visualized using an ECL detection kit.

### Immunohistochemistry analysis

Immunohistochemistry analysis was performed as described previously^18^ with a modification. Immunohistochemistry was performed on 4% paraformaldehyde-fixed buffalo (*Bubalus bubalis*) testes. The paraffin-embedded testicular sections were dewaxed, dehydrated, and endogenous peroxidase activity was quenched by methanol containing 3% H_2_O_2_ for 30 min. Sections were subjected to antigen retrieval in 0.01 M sodium citrate buffer (pH 6.0) using microwave heating. The sections were then incubated in blocking solution (5% BSA) for 2 h at room temperature and incubated overnight at 4 ℃ with primary antibody against NUCKS1 (bs-7917R, Bioss Biotechnology Inc.), LMNA (phospho S392) (ab58528, Abcam), and PSMA3 (phospho S250) (bs-9353R, Bioss Biotechnology Inc.) at a dilution of 1:200. After washing twice in PBS-Tween-20, the sections were incubated with secondary antibody labeled with FITC (ab6717, Abcam, 1:200) for 1 h at room temperature, and then covered with a Prolong Gold antifade reagent with DAPI (Life Technologies, New York, USA) and kept in the dark until photographed using an Olympus IX73 inverted fluorescence microscope (Olympus, Tokyo, Japan). The negative control was incubated in blocking solution without the primary antibody.

### Statistical analysis

Comparisons between groups were evaluated using one-way analysis of variance (ANOVA). All quantitative data were presented as means ± standard error of the mean from at least three independent experiments, and a probability of P ≤ 0.05 was considered to be statistically significant.

## Supplementary information

Supplementary Information 1.

Supplementary Information 2.
