# Supplementary material for: Novel targets identified by integrated proteomic and phosphoproteomic analysis in spermatogenesis of swamp buffalo (Bubalus bubalis)
Source: Sci Rep. 2020 Sep 24;10:15659. doi: 10.1038/s41598-020-72353-4 (PMC7515895; doi:10.1038/s41598-020-72353-4)
Supplement: Supplementary file 1 — Supplementary Information 1. [file 41598_2020_72353_MOESM1_ESM.docx]

**Legends to** **supplementary files**

**Supplementary Fig.S1. Tight junction pathway^53^.**

**Supplementary Fig.S2. Spliceosome pathway^53^.**

**Supplementary Fig.S3. Full-length gels of ODF1, NUCKS1, LMNA (pS392), PSMA3 (pS250) and β-actin.**

**References:**

**53. Kanehisa, M. and Sato, Y.; KEGG Mapper for inferring cellular functions from protein sequences. *Protein Science* 29, 28-35 (2019)**
