# Supplementary material for: Novel targets identified by integrated proteomic and phosphoproteomic analysis in spermatogenesis of swamp buffalo (Bubalus bubalis)
Source: Sci Rep. 2020 Sep 24;10:15659. doi: 10.1038/s41598-020-72353-4 (PMC7515895; doi:10.1038/s41598-020-72353-4)
Supplement: Supplementary file 2 — Supplementary Information 2. [file 41598_2020_72353_MOESM2_ESM.zip › supplementary files/Title page.docx]

**Novel targets identified by integrated proteomic and phosphoproteomic analysis in spermatogenesis of swamp buffalo (*Bubalus bubalis*)**

Yu-lin Huang^1#^, Peng-fei Zhang^2#^, Qiang Fu^2^, Weng-tan He^2^, Kai Xiao^2^, Ming Zhang^2*^

^1^ *Department of Cell and Genetics, College of Basic Medicine,* *Guangxi University of Chinese Medicine, Nanning, Guangxi, China*

^2^ *State Key Laboratory for Conservation and Utilization of Subtropical* *Agro-Bioresources,* *Animal Reproduction Institute, Guangxi University, Nanning, Guangxi, China*

*Corresponding author at:

Ming Zhang, State Key Laboratory for Conservation and Utilization of Subtropical Agro-Bioresources, Animal Reproduction Institute, Guangxi University, Nanning, Guangxi, China. Email: [mingzhang@gxu.edu.cn](mailto:mingzhang@gxu.edu.cn), Tel: +86 13877163681

^#^ Yu-lin Huang and Peng-fei Zhang contributed equally to this work.
