# Supplementary figures and images for: Novel targets identified by integrated proteomic and phosphoproteomic analysis in spermatogenesis of swamp buffalo (Bubalus bubalis)
Source: Sci Rep. 2020 Sep 24;10:15659. doi: 10.1038/s41598-020-72353-4 (PMC7515895; doi:10.1038/s41598-020-72353-4)

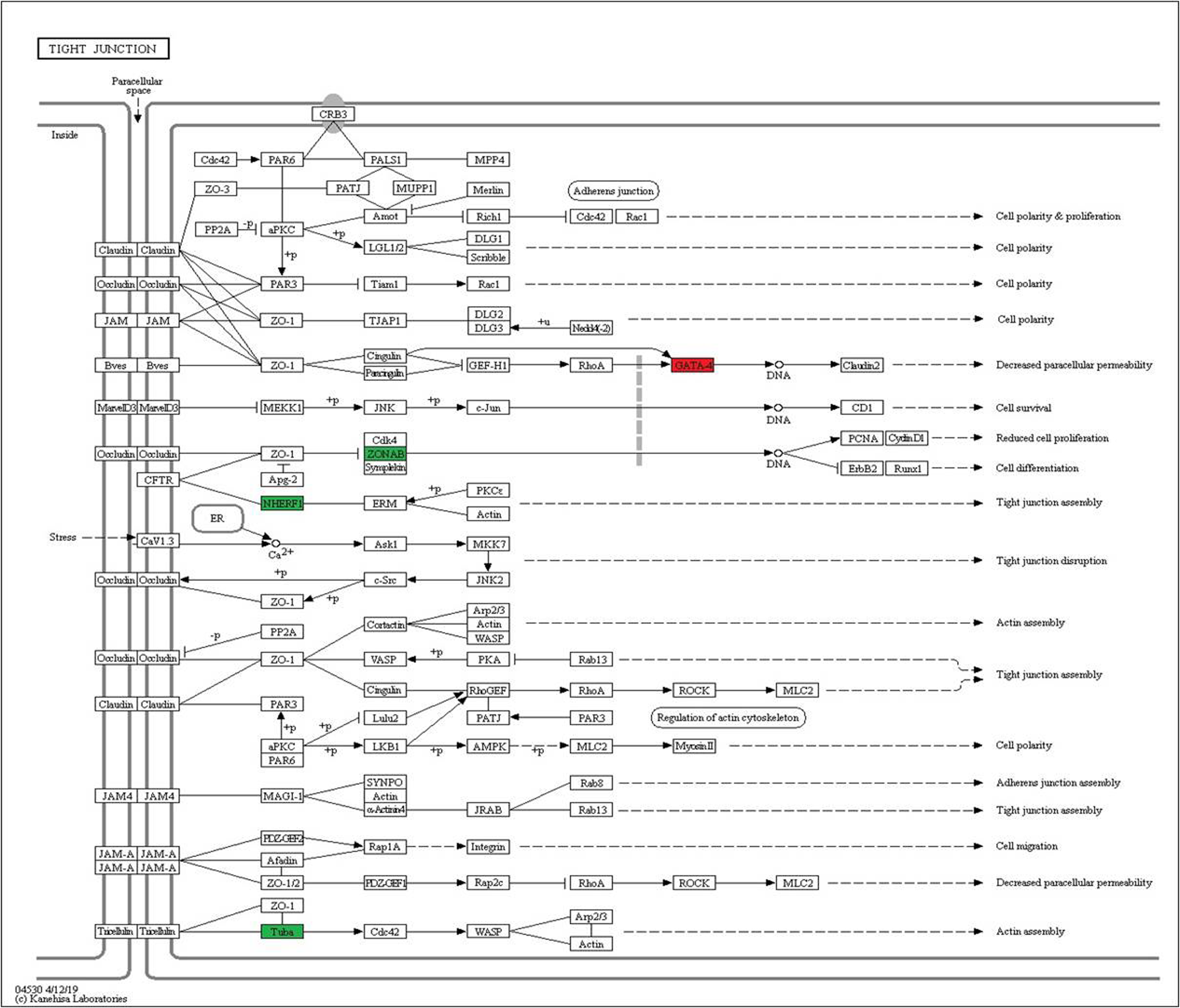

Supplement: Supplementary file 2 — Supplementary Information 2. [file 41598_2020_72353_MOESM2_ESM.zip › supplementary files/Supplementary Figure S1.tif]

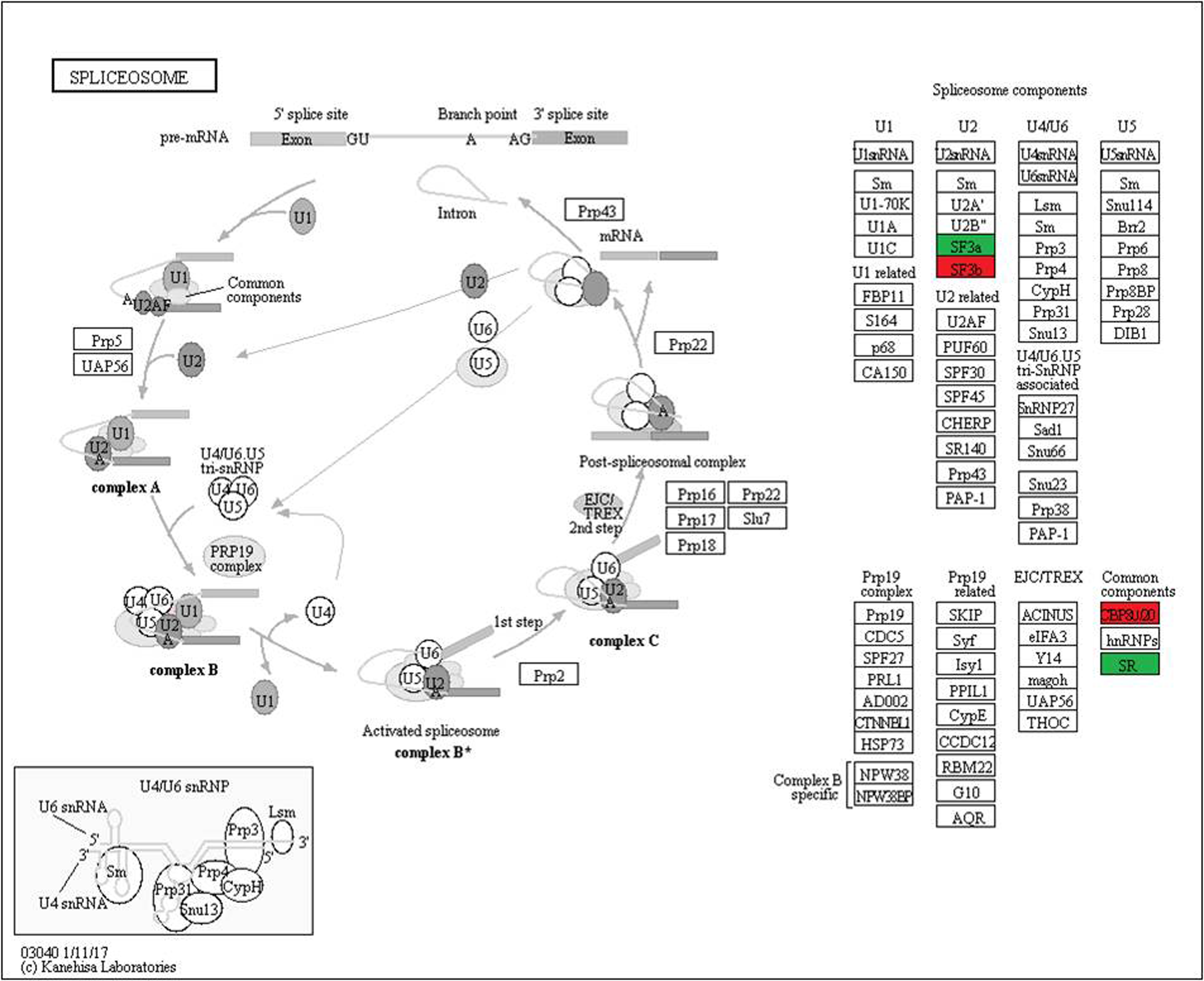

Supplement: Supplementary file 2 — Supplementary Information 2. [file 41598_2020_72353_MOESM2_ESM.zip › supplementary files/Supplementary Figure S2.tif]

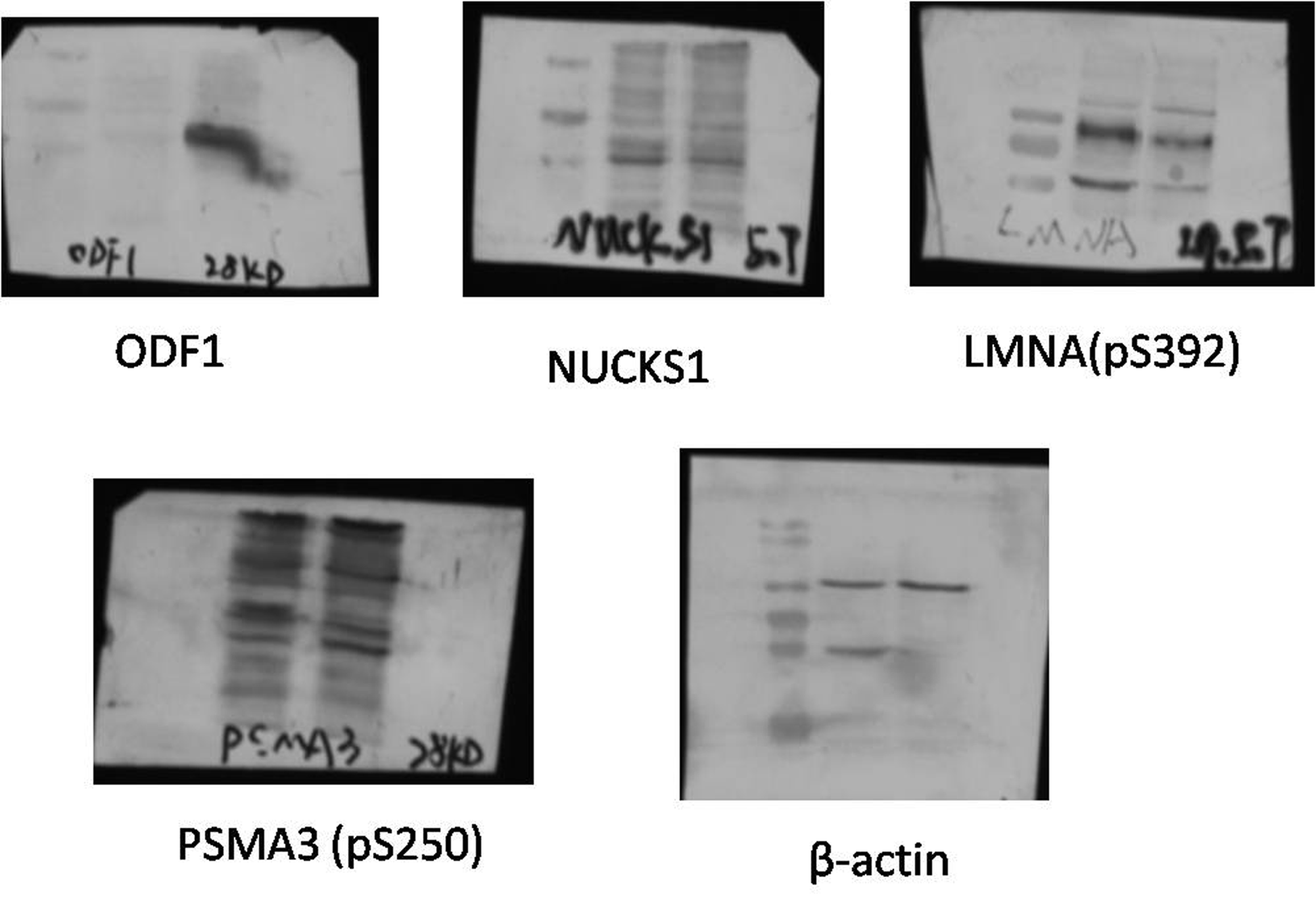

Supplement: Supplementary file 2 — Supplementary Information 2. [file 41598_2020_72353_MOESM2_ESM.zip › supplementary files/Supplementary Figure S3.tif]
